# The Influence of Acetaminophen on Task Related Attention

**DOI:** 10.3389/fnins.2019.00444

**Published:** 2019-05-03

**Authors:** Sumeet Mutti Jaswal, Javier A. Granados Samayoa, Julia W. Y. Kam, Daniel Randles, Steven J. Heine, Todd C. Handy

**Affiliations:** ^1^Department of Psychology, The University of British Columbia, Vancouver, BC, Canada; ^2^Department of Psychology, Ohio State University, Columbus, OH, United States; ^3^Department of Psychology, University of California, Berkeley, Berkeley, CA, United States; ^4^Department of Psychology, University of Toronto Scarborough, Toronto, ON, Canada

**Keywords:** acetaminophen, mind wandering, ERP, attention, default mode network, salience network

## Abstract

Our study was designed to examine whether the pain reliever acetaminophen impacts the normal ebb-and-flow of off-task attentional states, such as captured by the phenomenon of mind wandering. In a placebo-controlled between-groups design, participants performed a sustained attention to response task while event-related potentials (ERPs) to target events were recorded. Participants were queried at random intervals for their attentional reports – either “on-task” or “off-task.” The frequency of these reports and the ERPs generated by the preceding target events were assessed. Behaviorally, the frequency of off-task attentional reports was comparable between groups. Electrophysiologically, two findings emerged: first, the amplitude of the P300 ERP component elicited by target events was significantly attenuated during off-task vs. on-task attentional states in both the acetaminophen and placebo groups. Second, the amplitude of the LPP ERP component elicited by target events showed a significant decrease during off-task attentional states that was specific to the acetaminophen group. Taken together, our findings support the conclusion that acetaminophen doesn’t impact our relative propensity to drift into off-task attentional states, but it does affect the depth of neurocognitive disengagement during off-task attentional states, and in particular, at the level of post-categorization stimulus evaluations indexed by the LPP.

## Introduction

Acetaminophen—also known as paracetamol or Tylenol^®^—is a common non-prescription, centrally-acting pain reliever that has been recognized in recent years to have a number of substantive cognitive and affective side-effects. In the original study on the topic it was found that social pain was attenuated by chronic doses of acetaminophen taken over a period of 3 weeks (DeWall et al., 2010). Since then, the side effects of acetaminophen have been expanded on in a number of different ways, all demonstrating that it attenuates various aspects of cognitive-affective processes — including reduced emotional reactions to affectively polarized images (Durso et al., 2015); reduced social anxiety (Fung and Alden, 2017); reduced affective reactions to negative events (DeWall et al., 2015); reduced empathy (Mischkowski et al., 2016); reduced distrust of others (Roberts et al., 2018); reduced affective responses to cognitively threatening events (Randles et al., 2013); and reduced implicit analysis of behavioral errors (Randles et al., 2016).

Why might acetaminophen induce such effects? The available evidence from functional neuroimaging has demonstrated that acetaminophen modulates responsivity in the dorsal anterior cingulate cortex (dACC) during tasks invoking both social (DeWall et al., 2010) and physical pain (Pickering et al., 2015). Given that dACC function has been tied to behavioral performance monitoring (Holroyd et al., 2004; Brown and Braver, 2005; Sheth et al., 2012) and the evaluative analysis of behaviorally-relevant events (Bush et al., 2002; Blair et al., 2006; Cai and Padoa-Schioppa, 2012; Kolling et al., 2012), acetaminophen’s attenuating effects on reactivity to behavioral errors, poor decision outcomes, emotionally-charged stimuli, social rejection and the like (Randles et al., 2013, 2016; DeWall et al., 2015, 2010; Mischkowski et al., 2016) all functionally align with this possibility.

At the same time, the dACC has also been implicated in modulating the depth of our attentional engagement with the external task environment (Heilbronner and Hayden, 2016), and this is the point of focus in our study. In particular, the dACC has been identified as one of two cortical hubs in the brain’s “salience network” (Sridharan et al., 2008; Menon and Uddin, 2010), a network which is believed to control the depth of our attention to the external task environment through its modulatory influence on the brain’s default mode network (or DMN). Specifically, when our attention transiently drifts away from the given task at hand such as during periods of mind wandering, it correlates with a systematic up-regulation of activity in the DMN (Mason et al., 2007; Christoff et al., 2009; Andrews-Hanna et al., 2010; Andrews-Hanna, 2012). This has supported the idea that DMN activation is associated with spontaneous, task-independent thought (Christoff et al., 2016; Dixon et al., 2017). Against this background, the salience network has been shown to transiently suppress activity in the DMN, leading to the proposal that this modulatory influence maintains “on-task” attentional states because the neural circuitry associated with “off-task” attentional states — the DMN — is being directly inhibited (Bonnelle et al., 2012; Jilka et al., 2014; Menon, 2015).

Given the collective evidence, our study thus addressed the following question: might acetaminophen impact the normal ebb and flow of on-task vs. off-task attentional states? We suggest that the question warrants consideration for two central reasons.

First, when our thoughts drift or wander away from the on-going task at hand, there is a transient but widespread attenuation in the cortical processing of external events during the off-task attentional state that extends across the sensory-perceptual (Kam et al., 2011; Smallwood et al., 2011; Baird et al., 2014), cognitive (Smallwood et al., 2008; Barron et al., 2011; Kam et al., 2013), affective (Kam et al., 2014), and motor/performance-monitoring (Kam et al., 2012) domains. Such effects are believed to reflect a basic functional tension that persists in the brain between prioritizing external vs. internal inputs for processing by capacity-limited resources over time, as we naturally vacillate between on-task and off-task attentional states (Smallwood et al., 2011; Kam and Handy, 2013; Handy and Kam, 2015). In light of this, if acetaminophen does in fact alter the normal ebb and flow between on-task vs. off-task attentional states — such as by increasing the relative proportion of time spent in off-task attentional states, and/or by increasing the depth of our processing attenuation during off-task attentional states — it raises potential health and safety concerns. For example, falling in older individuals, a leading cause of death and disability in seniors, is associated with higher rates of mind wandering (Nagamatsu et al., 2013).

Second, taken together, the neurocognitive and neuroaffective side effects of acetaminophen as reviewed above have supported the hypothesis that the drug influences the brain’s social-affective evaluative processes (DeWall et al., 2015). While our intention here is not to inform on the validity of this hypothesis, if acetaminophen is found to alter normative patterns of on- vs. off-task attentional states, it would represent an important expansion in our basic understanding of acetaminophen and the breadth of its neurocognitive effects. That is, to our knowledge there has been no evidence reported to date indicating that acetaminophen alters attentional processes such as those associated with visual target identification, for example, processes impacted by off-task attentional states (Smallwood et al., 2008; Barron et al., 2011; Kam et al., 2013) that precede more evaluative stages of analyses in the afferent stream of visual stimulus processing.

To address our primary question, we combined a canonical event-related potential (ERP) paradigm we have used previously for assessing on- vs. off-task attentional states (Smallwood et al., 2008; Kam et al., 2011) with a between-group, double-blind, placebo-controlled procedure for administering acetaminophen that we have also used previously, both with (Randles et al., 2016) and without (Randles et al., 2013) ERPs as a dependent measure. Participants were asked to perform a simple visual target detection task while we recorded their ERP responses to target events. At random intervals they were then stopped and prompted to report on their attentional state just prior to the stoppage—either “on-task” vs. “off-task.” Participants in the “acetaminophen” condition consumed a 1000 mg capsule of acetaminophen approximately 1 h prior to the initiation of data collection, while participants in the “placebo” condition consumed an identical capsule containing granulated sugar. Our primary dependent measures were (1) the relative frequencies of on- vs. off-task attentional reports, to assess acetaminophen’s impacts on the relative proportion of time spent in off-task attentional states, and (2) the ERPs elicited by target events, to assess acetaminophen’s possible impacts on the depth of our attentional and evaluative processing attenuation during off-task attentional states, as indexed by the P300 and LPP ERP components respectively.

## Materials and Methods

### Participants

Participants were recruited through the community via posting at the Paid Participants Study List hosted by Psychology Graduate Student Council website, and remunerated $20 (CAD) for their participation. A total of 60 participants from the community were recruited, but data from 20 participants was excluded from final analyses due to excessive eye movement and other recording artifacts identified in their data during initial data analysis (see below). Of the remaining forty participants (26 females; *M* = 23.4 years old, *SD* = 6.61; 39 were right-handed), all had no history of neurological problems, and had normal or corrected-to-normal vision. In the placebo group, participants had a mean age of 23.9 (*SD* = 6.77; 15 females), and in the acetaminophen group, the mean age was 22.9 (*SD* = 6.57; 11 females). Participants were asked to not participate if they had a history of alcoholism or consume on average more than two alcoholic drinks a day, were a smoker, were pregnant or breastfeeding, were asthmatic or diabetic, had been diagnosed with liver, stomach, gastrointestinal, heart, or kidney disease or an allergy or family history of allergy to Tylenol (acetaminophen), had experienced intracranial bleeding or had a tendency to bleed excessively, or were at the time of participation taking any medication other than oral contraceptives. We did not assess the participants’ daily pain medication use. However, when participants’ alcohol, cigarette, and cannabis use over the past 24 h was assessed, we found no significant difference between the two experimental groups (*p* ≥ 0.15). Participants provided written informed consent to the experimental procedure, and the UBC Behavioral Review Ethics Board approved all procedures and protocols of this experiment.

### Stimuli and Task

**FIGURE 1:**
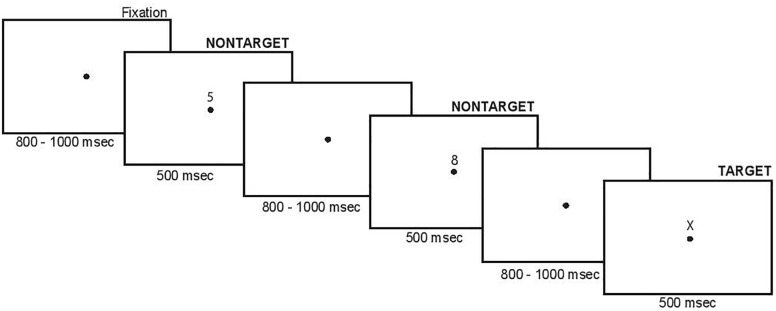
Timing and sequence of stimuli in the present study.

Participants performed a SART adapted from Smallwood et al. (2008) and Kam et al. (2011). The task involved presentation of a serial stream of stimuli at foveal centered fixation dot. The stimuli were black numbers or a letter on a white background. Participants were asked to make a manual button press for “non-targets” (numbers 0–9, which were presented frequently), and were asked to withhold a button press response when presented with a “target” (letter X, which was presented infrequently). The timing and sequence of stimuli are shown in Figure 1. Within each block of stimuli, target probability was quasi-randomized, with the constraints that (1) one to two targets were presented during each block, and (2) for blocks having two targets, the targets would be separated by at least 10 non-target events, and (3) the last six trials of each block included no target events. The block duration was randomly varied between 30 and 90 s, meaning each block had anywhere from 15 to 45 trials, and each participant completed a minimum of 20 blocks in total.

To measure task-related attention, participants were instructed to report their “attentional state” at the end of each trial block. Specifically, they were asked to identify their state immediately prior to the block termination as either being “on-task” (fully attentive to task performance), or “off-task” (inattentive to the task). Importantly, participants were provided with verbal descriptions and examples of these two “attentional states” prior to starting the testing session. On-task states were defined as when one’s attention is firmly directed toward the task, whereas off-task states were described as when one is aware of other things than just the task at hand. Examples of these attentional states were given in the context of reading, during which “one may be fully attentive to the content of the reading material, or thinking about something completely unrelated to the content, reflective of on-task and off-task states, respectively.” Attentional reports were recorded by the investigator at the conclusion of each trial block, and these reports were then used to sort ERP data based on on-task versus off-task states as described below. The block duration itself was randomly varied between 30 and 90 s to (1) minimize predictability of block completion and (2) maximize variability of attentional state at the time of block completion.

### Experimental Manipulation

Acetaminophen is a widely used non-opiate, analgesic, and antipyretic drug, that is believed to impact the brain in complex ways. Comparably to non-steroidal anti-inflammatory drugs (NSAID), acetaminophen suppresses prostaglandin synthesis by the inhibitory effect on COX-1 and COX-2 activity (Ouellet and Percival, 2001; Graham and Scott, 2005). However, unlike NSAIDs, acetaminophen produces weak anti-inflammatory effects due to its lack of significant influence on the peripheral COX enzymes (Clissold, 1986). Acetaminophen when studied in combination with 5-HT_3_ receptors demonstrates stimulation of the descending serotoninergic pathway (Pickering et al., 2008). Moreover, acetaminophen is regarded as a prodrug that due to its active metabolization results in a fatty acid amid (*N*-arachidonoyl phenol amine; AM404) that indirectly increases activity in the endocannabinoid system (Högestätt et al., 2005). Thus, the pain sensation of the tissue receptors through the spinal cord to the thalamus and the cerebral cortex are impacted by acetaminophen (Jóźwiak-Bebenista and Nowak, 2014). Our procedures for the between-group pharmacological manipulation replicated Randles et al. (2016) such that participants in the experimental condition consumed two capsules each containing 500 mg tablets of Kirkland-brand acetaminophen (1000 mg total), while participants in the placebo condition consumed two identical-looking capsules that were filled with white granulated sugar. Participants were informed that they were being randomly assigned to an experimental condition through double-blind procedure, where each participant’s dose was assigned a unique ID prior to the study that matched it to the correct condition. While the researcher running the study was blind to condition assignment, they could request access to identify the condition if it became medically necessary. Participants were sorted into their assigned condition beginning at the group averaging stage of data analysis. The testing took place between 10 am and 7 pm, and there was no significant difference when comparing the two experimental groups for the time the testing took place [*t*(38) = 0.82, *p* = 0.42].

### Procedure

Participants provided written consent and then consumed their assigned capsules. The participant was then prepared for recording his or her electroencephalogram (EEG), and instructed in the SART while seated at the task computer running MATLAB R2010a (The MathWorks, Inc.) with Psychtoolbox. This task began approximately 60 min after consuming the pills, ensuring that most participants experienced peak pharmacological activation; typically requiring 45–60 min for adults consuming acetaminophen orally (Bertolini et al., 2006). The half-life of acetaminophen in healthy subjects has been reported to be 1.9 to 2.5 h (Forrest et al., 1982). Once commenced, the EEG testing session itself took approximately 1 h, giving participants a total study time of approximately 2 h.

### Electrophysiological Recording and Analysis

Continuous EEG was recorded during the task via 64 Ag/AgCl active electrodes mounted in an elastic cap (BioSemi Active-Two amplifier system; BioSemi, Amsterdam, Netherlands) in spatial accordance with the international 10–20 system. Two additional electrodes located over the medial-parietal cortex (Common Mode Sense and Driven Right Leg) were used as ground electrodes. Recordings were digitized at 256 Hz, digitally filtered offline between 0.1 and 30 Hz (zero phase-shift Butterworth filter) and then referenced offline to the average of two mastoid electrodes. EEG data processing was performed using ERPLAB, a toolbox within MATLAB 2012a (The MathWorks, Inc.) used in conjunction with EEGLAB. To ensure proper eye fixation and allow for the removal of events associated with eye movement artifacts, vertical and horizontal electrooculograms (EOGs) were also recorded—the vertical EOGs from an electrode inferior to the right eye, and the horizontal EOGs from two electrodes on the right and left outer canthus. Offline, computerized artifact rejection was used to eliminate trials during which detectable eye movements and blinks occurred. These eye artifacts were detected by identifying the minimum and maximum voltage values on all recorded EOG channels from −200 to 800 ms post-stimulus for each event epoch, and then removing the trial from subsequent signal averaging if that value exceeded 200 —V, a value calibrated to capture all blinks and saccades. This was followed by visual inspection of the data. If additional artifacts (e.g., muscle movements and loose connections) were observed, the threshold was reduced by 25 —V until artifacts were not present or a minimum of 100 —V was reached. Participants (*N* = 20) with more than 50% rejected trials were excluded from analysis; the remainder of participants (*N* = 40) had an average of 17.84% trials rejected due to these signal artifacts; the percentage of rejected trials did not significantly differ between individuals in the acetaminophen vs. placebo conditions (*p* = 0.53), and the mean number of artifact-free trials retained in each experimental group and attentional state are reported in Table 1. Of the removed participants, 10 were in the acetaminophen experimental group.

**Table 1:** Artifact-free trials.

	Attention State	
	On-Task	Off-Task
Placebo	86.60 (41.65)	68.25 (29.98)
Acetaminophen	84.27 (37.84)	88.40 (43.78)

*The mean number of artifact-free trials retained as a function of attentional state (on task vs. mind wandering) and experimental group (acetaminophen vs. placebo). Standard deviations are in parentheses.*

The ERP waveforms for the two attentional conditions of interest were derived by averaging together the EEG epochs for the six non-target events preceding each categorical instance of a subjective attentional report (on-task vs. off-task). Although it is never certain how long participants have actually been in a particular attentional state at the time a subjective report is given, analyses were based on the assumption that the 12 s prior to each report (the time window capturing the six preceding non-targets) would, on average, reliably capture the given attentional state—an assumption consistent with the presumed time course of off-task thinking (e.g., Sonuga-Barke and Castellanos, 2007; Christoff et al., 2009) and the time windows of analyses we have adopted previously (Smallwood et al., 2008; Kam et al., 2011, 2012; Kirschner et al., 2012; Kam and Handy, 2013). Although a shorter pre-report time window for averaging non-target EEG epochs would more accurately capture attentional state, it would also reduce the number of events included in the ERP analysis. The choice of how many pre-report events to include in the averages was therefore an attempt to maximize the number of events in each waveform average while not extending the window back so far in time as to consistently capture the preceding attentional state or transition between states.

## Results

As outlined above, the goal of our study was to examine whether acetaminophen disrupts the normal ebb and flow of on-task vs. off-task attentional states. Our primary dependent measures were (1) the relative frequencies of on- vs. off-task attentional reports, to assess acetaminophen’s impacts on the relative proportion of time spent in on- vs. off-task attentional states, and (2) the ERPs elicited by target events, to assess acetaminophen’s impacts on the depth of our attentional and evaluative processing attenuation during off-task attentional states, as indexed by the mean amplitudes of the P3 and LPP ERP components elicited by target events, respectively.

### Attentional Reports

Overall, participants completed an average of 36.2 trial blocks during the 1-h testing session, with 49.4% of the attentional reports being given as “off-task.” However, there was no significant between-group difference in the reported frequency of “off-task” attentional states [acetaminophen = 51.20%, placebo = 47.85%; *t*(38) = 0.59, *p* = 0.56].

### Event-Related Potentials

Analyses of the ERPs elicited by the non-target events in the 12 s immediately preceding each attentional report focused on two components of interest: the P300, which increases in amplitude with the degree of attention allocated to the ERP-eliciting stimulus (Donchin, 1981; Polich, 2007), and the late positive potential (or LPP) that follows the P3 in time, which increases in amplitude with the degree of post-attentional stimulus evaluation (e.g., Cacioppo and Berntson, 1994; Crites et al., 1995; Cacioppo et al., 1996; Cuthbert et al., 2000; Ito and Cacioppo, 2000) even for implicit evaluative analyses (Handy et al., 2010). The P300 was chosen *a priori* for analysis because previous research has demonstrated that the amplitude of the P300 elicited by task-relevant events systematically decreases during off-task attentional states (Smallwood et al., 2008; Barron et al., 2011); at issue in the current study was whether acetaminophen might increase the depth of *attentional* disengagement during off-task attentional states, as indexed by the P300. Likewise, the LPP was chosen *a priori* for analysis because acetaminophen has been associated with reductions in the evaluative analysis of external events (DeWall et al., 2010, 2015; Randles et al., 2013, 2016; Mischkowski et al., 2016); at issue in the current study was whether there might be an increase in the depth of this *evaluative* disengagement during off-task attentional states. For both ERP components, the analyses reported below were all based on mean amplitude measures centered on the approximate peak of the component of interest as identified in the group-averaged waveforms, measured relative to a −200 to 0 ms pre-stimulus baseline.

### Attentional Disengagement: P300

**FIGURE 2:**
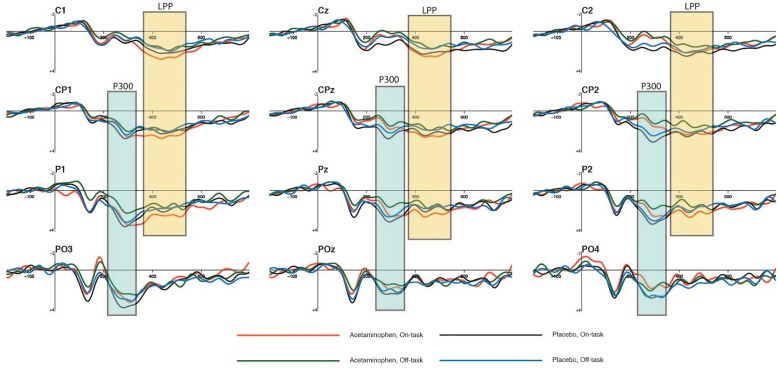
Averaged waveforms showing cognitive effects of P300 and LPP ERP components elicited by non-targets at 12 electrode sites as a function of attentional state and experimental condition. The time-windows used in the P300 amplitude analyses are highlighted in blue (250–350 ms), and the ones used in the LPP amplitude analyses are highlighted in yellow (375–525 ms). These ERP waveforms are time-locked to visual non-target events in the 12 s preceding an attentional report.

The P300 elicited by non-target events is shown in Figure 2 at the midline-posterior scalp electrode locations CPz, Pz, and POz where it was maximal in amplitude, along with the immediately distal locations to each over the left (CP1, P1, and PO1) and right (CP2, P2, and PO2) cerebral hemispheres. The mean amplitude of the P300 at each of these electrode sites is reported in Table 2 as a function of attentional report (on-task vs. off-task) and experimental condition (acetaminophen vs. placebo), as measured across a 250 to 350 ms time window capturing the approximate P300 peak. It appeared that there was a general reduction in P300 amplitude during off-task relative to on-task attentional states, but no impact of acetaminophen on this effect. This data pattern was confirmed by an omnibus, repeated-measures ANOVA that included experimental group (acetaminophen vs. placebo) as a between-groups factor, and attentional report (on-task vs. off-task) and electrode site included as within-groups factors. There was an overall significant main effect of attentional report [*F*(1,38) = 4.64, *p* = 0.038, $\eta_{p}^{2}$ = 0.11] with an overall greater amplitude during on-task attentional reports, but there was no significant main effect of experimental group [*F*(1,38) = 2.22, *p* = 0.14] or a significant interaction between attentional report and experimental group [*F*(1,38) = 0.11, *p* = 0.75]. As well, although a main effect of electrode site indicated an overall difference in P300 amplitude across scalp electrode site [*F*(8,31) = 6.38, *p* < 0.001], there was no significant interaction between electrode site and experimental group [*F*(8,31) = 1.28, *p* = 0.29], or interaction of electrode site with attentional state [*F*(8,31) = 1.15, *p* = 0.36].

**Table 2:** P300 ERP results.

*P300*		Attention State	
Condition	Electrodes	On-Task	Off-Task
Placebo	CP1	2.02 (1.42)	1.67 (1.48)
	CPz	2.03 (1.23)	1.63 (1.78)
	CP2	2.36 (1.36)	1.91 (1.70)
	P1	2.74 (1.07)	2.38 (1.50)
	Pz	2.40 (1.28)	2.00 (1.70)
	P2	2.59 (1.34)	2.33 (1.74)
	PO3	3.01 (1.66)	2.43 (1.90)
	POz	1.99 (1.36)	1.75 (1.61)
	PO4	2.24 (1.25)	2.24 (1.42
Acetaminophen	CP1	1.93 (1.53)	1.48 (1.15)
	CPz	1.33 (1.96)	0.99 (1.40)
	CP2	1.24 (2.01)	0.82 (1.54)
	P1	2.68 (2.96)	1.66 (1.46)
	Pz	1.91 (2.16)	1.14 (1.67)
	P2	1.96 (1.96)	1.33 (1.77)
	PO3	2.36 (1.97)	2.03 (1.57)
	POz	1.45 (2.21)	1.22 (1.72)
	PO4	1.35 (3.63)	1.42 (2.01)

*The mean P300 amplitudes (and standard deviation) for non-targets as a function of attentional state (on task vs. mind wandering) and experimental group (acetaminophen vs. placebo). Mean amplitudes were taken across a 250–350 ms post-stimulus time window, measured relative to a −200 to 0 baseline.*

### Evaluative Disengagement: LPP

The LPP elicited by non-target events is shown in Figure 2 over the midline-posterior scalp electrode locations where it was maximal (Cz, CPz, and Pz) along with the immediately distal locations to each over the left (C1, CP1, and P1) and right (C2, CP2, and P2) cerebral hemispheres. The mean amplitude of the LPP at each of these electrode sites is reported in Table 3 as a function of attentional report (on-task vs. off-task) and experimental condition (acetaminophen vs. placebo), as measured across a 375 to 525 ms time window capturing the approximate LPP maximum. Like the P300, there appeared to be a general reduction in LPP amplitude during off-task relative to on-task attentional states, but unlike the P300, this effect also appeared to be greater in the acetaminophen relative to placebo group. This data pattern was confirmed via an omnibus, repeated-measures ANOVA that included experimental group (acetaminophen vs. placebo) as a between-groups factor, and attentional report (on-task vs. off-task) and electrode site as within-groups factors. Specifically, there was an overall significant main effect of attentional report [*F*(1,38) = 21.66, *p* < 0.001, $\eta_{p}^{2}$ = 0.36] and a significant group × attentional report interaction [*F*(1,38) = 5.84, *p* = 0.021, $\eta_{p}^{2}$ = 0.13], but there was no significant main effect of group [*F*(1,38) = 0.13, *p* = 0.91]. Separate ANOVAs within each group confirmed a main effect of attentional report for the acetaminophen group [*F*(1,38) = 25.00, *p* < 0.001, $\eta_{p}^{2}$ = 0.40], but not for the placebo group [*F*(1,38) = 2.50, *p* = 0.12]. However, separate between-groups comparisons within each attentional condition indicated that there was no significant between-group effect in either the on-task [*F*(1,38) = 0.53, *p* = 0.47] or off-task attentional condition [*F*(1,38) = 0.27, *p* = 0.61]. As well, there was also no significant main effect of electrode site [*F*(8,31) = 1.44, *p* = 0.22], or interaction of electrode site with experimental group [*F*(8,31) = 1.32, *p* = 0.27], or interaction of electrode site with attentional state [*F*(8,31) = 0.97, *p* = 0.48].

**Table 3:** LPP ERP results.

*LPP*		Attention State	
Condition	Electrodes	On-Task	Off-Task
Placebo	C1	1.78 (1.43)	1.47 (1.37)
	Cz	1.78 (1.73)	1.33 (1.77)
	C2	1.92 (1.57)	1.63 (1.55)
	CP1	1.76 (1.14)	1.62 (1.20)
	CPz	1.87 (1.38)	1.52 (1.55)
	CP2	1.96 (1.15)	1.67 (1.36)
	P1	1.60 (1.11)	1.55 (1.11)
	Pz	1.54 (1.25)	1.37 (1.20)
	P2	1.54 (1.25)	1.52 (1.04)
Acetaminophen	C1	2.22 (1.63)	1.39 (1.27)
	Cz	2.00 (1.65)	1.19 (1.43)
	C2	1.74 (1.48)	1.23 (1.31)
	CP1	2.18 (1.40)	1.63 (1.29)
	CPz	2.11 (1.77)	1.41 (1.53)
	CP2	1.96 (1.55)	1.22 (1.41)
	P1	2.19 (2.07)	1.32 (1.21)
	Pz	2.00 (1.59)	1.24 (1.40)
	P2	1.98 (1.77)	1.22 (1.34)

*The mean LPP amplitudes (and standard deviation) for non-targets as a function of attentional state (on task vs. mind wandering) and experimental group (acetaminophen vs. placebo). Mean amplitudes were taken across a 375–525 ms post-stimulus time window, measured relative to a −200 to 0 baseline.*

### Additional Analyses

In addition to the above planned analyses, we conducted an additional set of analyses aimed at following up on two specific issues arising from our findings. First, given the significant interaction found between group and attentional state in the LPP data, we wanted to determine whether the impacts of acetaminophen might have also extended to behavioral performance. Second, given that one out of three participants had sufficient residual noise in their EEG data to preclude them from data analysis, we wanted to confirm that there were no systematic behavioral or demographic differences between the included vs. excluded participants that may have been biasing our data outcomes.

### Behavioral Performance

For assessing the hit rate for the non-targets button-presses, an omnibus, repeated-measures ANOVA, with experimental group (acetaminophen vs. placebo) as a between-groups factor, and attentional report (on-task vs. off-task) as within-groups factors was conducted. There was no significant main effect of experimental group [*F*(1,38) = 0.79, *p* = 0.38], or a significant main effect of attentional report [*F*(1,38) = 0.09, *p* = 0.77], or a significant interaction between attentional report and experimental group [*F*(1,38) = 0.006, *p* = 0.94]. We were not able to perform a false alarm analyses for incorrect button-presses for target events (“X”) in the 12 s preceding an attentional report, as these preceding trials were always non-targets (numbers).

For evaluating the reaction time for the button presses to non-targets, we conducted another omnibus, repeated-measures ANOVA, with experimental group as a between-groups factor, and attentional report as within-groups factors, and revealed no significant main effect of experimental group [*F*(1,38) = 0.10, *p* = 0.76], or a significant main effect of attentional report [*F*(1,38) = 1.92, *p* = 0.17], or a significant interaction between attentional report and experimental group [*F*(1,38) = 0.81, *p* = 0.37]. Participants were given a time of 1 s to respond following the non-target appearance to make a button-press response. Any button-presses that were made past this allotted time were thereby considered as a “miss” and recorded as a “0” in the accuracy analyses, and were omitted from the reaction time analyses.

For evaluating the reaction time variability for the button presses to non-targets, we conducted another omnibus, repeated-measures ANOVA, with experimental group as a between-groups factor, and attentional report as within-groups factors, and revealed a significant main effect of attentional report [*F*(1,34) = 4.68, *p* = 0.04, $\eta_{p}^{2}$ = 0.12]. A *post hoc* Tukey test revealed that participants varied significantly more when they reported being in off-task vs. on-task attentional state [*p* < 0.05]. However, there was no significant main effect of experimental group [*F*(1,34) = 1.14, *p* = 0.29], or a significant interaction between attentional report and experimental group [*F*(1,34) = 0.01, *p* = 0.92]. Note: there was omission of participants data that had only one or less reaction time during the experimental blocks as we were not able to assess their variability.

### Removed Participants

We also examined the 30% subject attrition from our analyses due to excessive ERP artifacts, to assess whether there was any meaningful difference between the subjects retained (*n* = 40) and the subjects removed (*n* = 19) on both demographic and behavioral information. We are unable to assess one participants’ data in our analyses, as they did not provide the researchers with consent for their data. For the remaining subjects, difference in age between the retained group of participants (*M* = 23.40, *SD* = 6.61), and the removed group of participants (*M* = 22.42, *SD* = 9.65) was non-significant [*t*(57) = 0.46, *p* = 0.65]. The retained group had a total of 26 females, whereas the removed group consisted of 16 females, however this group difference was not statistically significant [*t*(57) = −1.53, *p* = 0.13]. When comparing the retained and the removed groups on various behavioral analyses, there was no significant difference for the hit rate for button-presses to non-targets [*t*(57) = 0.43, *p* = 0.67], reaction time for button-presses to non-targets [*t*(57) = 0.69, *p* = 0.50], or false alarm rate for button-presses to target events [*t*(57) = 0.21, *p* = 0.83].

## Discussion

Our study examined the potential impacts of acetaminophen on off-task attentional states. Participants were randomly assigned to either an acetaminophen or placebo condition, and then performed a SART as they were queried on their attentional states and the ERPs elicited by non-target events were recorded. In terms of behavior, we found no significant differences in the frequency of off-task attentional reports for acetaminophen vs. placebo experimental groups. This suggests that acetaminophen did not influence the relative proportion of time spent in off-task attentional states. In terms of ERPs, there were two main findings of interest. First, there was an overall main effect of attentional state on the mean amplitude of the P300 elicited by non-target events, but no interaction in the P300 between attentional state and group. Given prior evidence showing that the P300 attenuates during off-task relative on on-task attentional states (Smallwood et al., 2008; Barron et al., 2011; Kam et al., 2011), this basic replication provided a critical measure of normative validity to our data.

More importantly however, there was a significant interaction between group and attentional state in the mean amplitude of the LPP elicited by non-target events, such that the LPP was significantly reduced in amplitude during off-task relative to on-task attentional states in the acetaminophen group, but no comparable attention effect was observed in the placebo group. This suggests that our null between-group effects in the frequency of attentional reports and the P300 amplitude cannot simply be ascribed to an ineffective dose of acetaminophen. Rather, our findings support the conclusion that acetaminophen can in fact impact the depth of neurocognitive disengagement during off-task attentional states, but that this effect appears to be restricted to the level of stimulus processing indexed by the LPP ERP component. Given the conclusions and findings, several key questions follow.

First, how should the differential effect of acetaminophen on the P300 vs. LPP be construed at a functional level? At issue here is understanding how these components differ in terms of what they are believed to capture in terms of neurocognitive processing. The P300 is taken to index the degree to which a stimulus is initially discriminated or categorized at a basic cognitive level (Donchin, 1981; Polich, 2007), while the LPP is believed to index deeper or more contemplative aspects of evaluative analysis (e.g., Cacioppo and Berntson, 1994; Crites et al., 1995; Cacioppo et al., 1996; Cuthbert et al., 2000; Ito and Cacioppo, 2000). For example, while the P300 modulates in amplitude with stimulus frequency (van Dinteren et al., 2014), the LPP modulates with emotional intensity (Brown et al., 2012) and implicit esthetic preference (Handy et al., 2010; Miller et al., 2011). Given this distinction, our findings here suggest that acetaminophen impacts neurocognitive disengagement during off-task attentional states not at the level of more basic stimulus categorization processes as indexed by the P300, but rather, at the level of post-categorization stimulus evaluation as indexed by the LPP. To be clear, however, whether this effect is restricted to implicit evaluative analysis as captured in the current paradigm, or whether it extends to situations where stimuli are being explicitly evaluated on emotional and/or esthetic dimensions remains an open question.

Second, how do our current findings extend our understanding of acetaminophen and its psychopharmacological side effects? As reviewed above, previous studies on the topic have converged on the conclusion that acetaminophen attenuates the evaluative analysis of affectively salient events. For example, individuals on acetaminophen present as less empathetic toward the physical plight of others (Mischkowski et al., 2016), less sensitive to the pain of social rejection (DeWall et al., 2010), less prone to cognitive dissonance with respect to behavioral outcomes (DeWall et al., 2015), less affected by existential threats (Randles et al., 2013), less reactive to emotionally valanced imagery (Durso et al., 2015), and less perturbed by performance errors (Randles et al., 2016). All of these situations can be described as having some degree of affective salience of one form or another. By way of contrast, the stimuli used in the current study were affectively neutral visual events presented in the context of a simple target detection task. Nevertheless, acetaminophen was found to induce a significant decrease in the implicit evaluative analysis of these stimuli, as captured in the LPP ERP component. This thus suggests that the psychological impacts of acetaminophen can extend to affectively benign stimuli. That is, the effects of acetaminophen on evaluative processing may not be specific to affectively-valenced stimuli and events.

Finally, to what extent if at all might our findings inform on the functional interactions between the putative site of acetaminophen’s effect in cortex — the dACC (DeWall et al., 2010; Pickering et al., 2015) — and the DMN, which upregulates in activity during off-task attentional states (Mason et al., 2007; Christoff et al., 2009; Andrews-Hanna et al., 2010; Andrews-Hanna, 2012)? To be certain, the ability to make neuroanatomically definitive statements in this regard go beyond the scope of what our study and methods allow. But given that qualifier, our findings do support the possibility that whatever the dACC’s role in the analysis of on-going external events (Heilbronner and Hayden, 2016), the evaluative functions impacted by acetaminophen (DeWall et al., 2015) may be functionally dissociable from those dACC processes involved in modulating DMN function (Bonnelle et al., 2012; Jilka et al., 2014; Menon, 2015). In other words, we did not find a significant impact of acetaminophen on the normal ebb-and-flow of off-task attentional states, or what would be predicted if acetaminophen were directly affecting DMN activation *per se*. Instead, using affectively neutral stimuli, we just found that acetaminophen’s oft-replicated propensity to attenuate the evaluative analysis of affectively-valenced events was more reliably present during off-task attentional states.

## Ethics Statement

This study was carried out in accordance with the recommendations of UBC Clinical Research Ethics Board with written informed consent from all subjects. All subjects gave written informed consent in accordance with the Declaration of Helsinki. The protocol was approved by the UBC Clinical Research Ethics Board.

## Author Contributions

JK, DR, SH, and TH contributed to the conception and design of the study. SJ and JGS acquired data for the work. JK helped with interpretation of data for the work. SJ drafted the manuscript and performed data analysis for the work. All authors contributed to manuscript revision, read and approved the submitted version.

## Conflict of Interest Statement

The authors declare that the research was conducted in the absence of any commercial or financial relationships that could be construed as a potential conflict of interest.

## References

[B1] Andrews-Hanna J. R. (2012). The brain’s default network and its adaptive role in internal mentation. *Neuroscientist* 18 251–270. 10.1177/1073858411403316 21677128 PMC3553600

[B2] Andrews-Hanna J. R. Reidler J. S. Sepulcre J. Poulin R. Buckner R. L. (2010). Article of the brain’s default network. *Neuron* 65 550–562. 10.1016/j.neuron.2010.02.005 20188659 PMC2848443

[B3] Baird B. Smallwood J. Lutz A. Schooler J. W. (2014). The decoupled mind: mind-wandering disrupts cortical phase-locking to perceptual events. *J. Cogn. Neurosci.* 26 2596–2607. 10.1162/jocn 24742189

[B4] Barron E. Riby L. M. Greer J. Smallwood J. (2011). Absorbed in thought: the effect of mind wandering on the processing of relevant and irrelevant events. *Psychol. Sci.* 22 596–601. 10.1177/0956797611404083 21460338

[B5] Bertolini A. Ferrari A. Ottani A. Guerzoni S. Tacchi R. Leone S. (2006). Paracetamol: new vistas of an old drug. *CNS Drug Rev.* 12 250–275. 10.1111/j.1527-3458.2006.00250.x 17227290 PMC6506194

[B6] Blair K. Marsh A. A. Morton J. Vythilingam M. Jones M. Mondillo K. (2006). Choosing the lesser of two evils, the better of two goods: specifying the roles of ventromedial prefrontal cortex and dorsal anterior cingulate in object choice. *J. Neurosci.* 26 11379–11386. 10.1523/JNEUROSCI.1640-06.2006 17079666 PMC6674525

[B7] Bonnelle V. Ham T. E. Leech R. Kinnunen K. M. Mehta M. A. Greenwood R. J. (2012). Salience network integrity predicts default mode network function after traumatic brain injury. *Proc. Natl. Acad. Sci. U.S.A.* 109 4690–4695. 10.1073/pnas.1113455109 22393019 PMC3311356

[B8] Brown J. W. Braver T. S. (2005). Learned predictions of error likelihood in the anterior cingulate cortex. *Science* 307 1118–1121. 10.1126/science.1105783 15718473

[B9] Brown S. van Steenbergen H. Band G. de Rover M. Nieuwenhuis S. (2012). Functional significance of the emotion-related late positive potential. *Front. Hum. Neurosci.* 6:33. 10.3389/fnhum.2012.00033 22375117 PMC3287021

[B10] Bush G. Vogt B. A. Holmes J. Dale A. M. Greve D. Jenike M. A. (2002). Dorsal anterior cingulate cortex: a role in reward-based decision making. *Proc. Natl. Acad. Sci. U.S.A.* 99 523–528. 10.1073/pnas.012470999 11756669 PMC117593

[B11] Cacioppo J. T. Berntson G. G. (1994). Relationship between attitudes and evaluative space: a critical review, with emphasis on the separability of positive and negative substrates. *Psychol. Bull.* 115 401–423. 10.1037//0033-2909.115.3.401

[B12] Cacioppo J. T. Crites S. L. Gardner W. L. (1996). Attitudes to the right: evaluative processing is associated with lateralized late positive event-related brain potentials. *Pers. Soc. Psychol. Bull.* 22 1205–1219. 10.1177/01461672962212002 16039143

[B13] Cai X. Padoa-Schioppa C. (2012). Neuronal encoding of subjective value in dorsal and ventral anterior cingulate cortex. *J. Neurosci.* 32 3791–3808. 10.1523/JNEUROSCI.3864-11.2012 22423100 PMC3319456

[B14] Christoff K. Gordon A. M. Smallwood J. Smith R. Schooler J. W. (2009). Experience sampling during fMRI reveals default network and executive system contributions to mind wandering. *Proc. Natl. Acad. Sci. U.S.A.* 106 8719–8724. 10.1073/pnas.0900234106 19433790 PMC2689035

[B15] Christoff K. Irving Z. C. Fox K. C. R. Spreng R. N. (2016). Mind-wandering as spontaneous thought: a dynamic framework. *Nat. Publish. Group* 17 718–731. 10.1038/nrn.2016.113 27654862

[B16] Clissold S. P. (1986). Paracetamol and Phenacetin. *Drugs* 32(Suppl. 4), 46–59. 10.2165/00003495-198600324-00005 3552585

[B17] Crites S. L. Cacioppo J. T. Gardner W. L. Berntson G. G. (1995). Bioelectrical echoes from evaluative categorization: II. A late positive brain potential that varies as a function of attitude registration rather than attitude report. *J. Pers. Soc. Psychol.* 68 997–1013. 10.1037/0022-3514.68.6.997 7608861

[B18] Cuthbert B. N. Schupp H. T. Bradley M. M. Birbaumer N. Lang P. J. (2000). Brain potentials in affective picture processing: covariation with autonomic arousal and affective report. *Biol. Psychol.* 52 95–111. 10.1016/S0301-0511(99)00044-7 10699350

[B19] DeWall C. N. Chester D. S. White D. S. (2015). Can acetaminophen reduce the pain of decision-making? *J. Exp. Soc. Psychol.* 56 117–120. 10.1016/j.jesp.2014.09.006

[B20] DeWall C. N. MacDonald G. Webster G. D. Masten C. L. Baumeister R. F. Powell C. (2010). Acetaminophen reduces social pain. *Psychol. Sci.* 21 931–937. 10.1177/0956797610374741 20548058

[B21] Dixon M. L. Andrews-hanna J. R. Spreng R. N. Irving Z. C. Mills C. Girn M. (2017). Interactions between the default network and dorsal attention network vary across default subsystems, time, and cognitive states. *Neuroimage* 147 632–649. 10.1016/j.neuroimage.2016.12.073 28040543

[B22] Donchin E. (1981). Surprise!. Surprise? *Psychophysiology* 18 493–513. 7280146 10.1111/j.1469-8986.1981.tb01815.x

[B23] Durso G. R. O. Luttrell A. Way B. M. (2015). Over-the-counter relief from pains and pleasures alike: acetaminophen blunts evaluation sensitivity to both negative and positive stimuli. *Psychol. Sci.* 26 750–758. 10.1177/0956797615570366 25862546 PMC4515109

[B24] Forrest J. A. H. Clements J. A. Prescott L. F. (1982). Clinical pharmacokinetics of paracetamol. *Clin. Pharmacokinet.* 7 93–107. 10.2165/00003088-198207020-00001 7039926

[B25] Fung K. Alden L. E. (2017). Once hurt, twice shy: social pain contributes to social anxiety. *Emotion* 17 231–239. 10.1037/emo0000223 27606825

[B26] Graham G. G. Scott K. F. (2005). Mechanism of action of paracetamol. *Am. J. Ther.* 12 46–55. 10.1097/00045391-200501000-00008 15662292

[B27] Handy T. C. Kam J. W. Y. (2015). Mind wandering and selective attention to the external world. *Can. J. Exp. Psychol.* 69 183–189. 10.1037/cep0000051 25844717

[B28] Handy T. C. Smilek D. Geiger L. Liu C. Schooler J. W. (2010). ERP evidence for rapid hedonic evaluation of logos. *J. Cogn. Neurosci.* 22 124–138. 10.1162/jocn.2008.21180 19199410

[B29] Heilbronner S. R. Hayden B. Y. (2016). Dorsal anterior cingulate cortex: a bottom-up view. *Annu. Rev. Neurosci.* 39 149–170. 10.1146/annurev-neuro-070815-013952 27090954 PMC5512175

[B30] Högestätt E. D. Jönsson B. A. G. Ermund A. Andersson D. A. Björk H. Alexander J. P. (2005). Conversion of acetaminophen to the Bioactive N-Acylphenolamine AM404 via fatty acid amide hydrolase-dependent arachidonic acid conjugation in the nervous system. *J. Biol. Chem.* 280 31405–31412. 10.1074/jbc.M501489200 15987694

[B31] Holroyd C. B. Nieuwenhuis S. Yeung N. Nystrom L. Mars R. B. Coles M. G. H. (2004). Dorsal anterior cingulate cortex shows fMRI response to internal and external error signals. *Nat. Neurosci.* 7 497–498. 10.1038/nn1238 15097995

[B32] Ito T. A. Cacioppo J. T. (2000). Electrophysiological evidence of implicit and explicit categorization processes. *J. Exp. Soc. Psychol.* 36 660–676. 10.1006/jesp.2000.1430

[B33] Jilka S. R. Scott G. Ham T. Pickering A. Bonnelle V. Braga R. M. (2014). Damage to the salience network and interactions with the default mode network. *J. Neurosci.* 34 10798–10807. 10.1523/JNEUROSCI.0518-14.2014 25122883 PMC4131006

[B34] Jóźwiak-Bebenista M. Nowak J. (2014). Paracetamol: mechanism of action, applications and safety concern. *Acta Pol. Pharm.* 71 11–23. 24779190

[B35] Kam J. W. Y. Dao E. Blinn P. Krigolson O. E. Boyd L. A. Handy T. C. (2012). Mind wandering and motor control: off-task thinking disrupts the online adjustment of behavior. *Front. Hum. Neurosci.* 6:329. 10.3389/fnhum.2012.00329 23248596 PMC3522104

[B36] Kam J. W. Y. Dao E. Farley J. Fitzpatrick K. Smallwood J. Schooler J. W. (2011). Slow fluctuations in attentional control of sensory cortex. *J. Cogn. Neurosci.* 23 460–470. 10.1162/jocn.2010.21443 20146593

[B37] Kam J. W. Y. Dao E. Stanciulescu M. Tildesley H. Handy T. C. (2013). Mind wandering and the adaptive control of attentional resources. *J. Cogn. Neurosci.* 25 952–960. 10.1162/jocn 23448525

[B38] Kam J. W. Y. Handy T. C. (2013). The neurocognitive consequences of the wandering mind: a mechanistic account of sensory-motor decoupling. *Front. Psychol.* 4:725. 10.3389/fpsyg.2013.00725 24133472 PMC3796327

[B39] Kam J. W. Y. Xu J. Handy T. C. (2014). I don’t feel your pain (as much): the desensitizing effect of mind wandering on the perception of others’ discomfort. *Cogn. Affect. Behav. Neurosci.* 14 286–296. 10.3758/s13415-013-0197-z 23900749

[B40] Kirschner A. Kam J. W. Y. Handy T. C. Ward L. M. (2012). Differential synchronization in default and task-specific networks of the human brain. *Front. Hum. Neurosci.* 6:139. 10.3389/fnhum.2012.00139 22661936 PMC3356872

[B41] Kolling N. Behrens T. E. J. Mars R. B. Rushworth M. F. S. (2012). Neural mechanisms of foraging. *Science* 336 95–98. 10.1126/science.1216930 22491854 PMC3440844

[B42] Mason M. F. Norton M. I. Van Horn J. D. Wegner D. M. Grafton S. T. Macrae C. N. (2007). Wandering minds: the default network and stimulus-independent thought. *Science* 315 393–396. 17234951 10.1126/science.1131295 PMC1821121

[B43] Menon V. (2015). “Salience network,” in *Brain Mapping: An Encyclopedic Reference* Vol. 2 ed. Toga A. W. (Amsterdam: Elsevier).

[B44] Menon V. Uddin L. Q. (2010). Saliency, switching, attention and control: a network model of insula function. *Brain Struct. Funct.* 214 655–667. 10.1007/s00429-010-0262-0 20512370 PMC2899886

[B45] Miller M. W. Rietschel J. C. McDonald C. G. Hatfield B. D. (2011). A novel approach to the physiological measurement of mental workload. *Int. J. Psychophysiol.* 80 75–78. 10.1016/j.ijpsycho.2011.02.003 21320552

[B46] Mischkowski D. Crocker J. Way B. M. (2016). From painkiller to empathy killer: acetaminophen (paracetamol) reduces empathy for pain. *Soc. Cogn. Affect. Neurosci.* 11 1345–1353. 10.1093/scan/nsw057 27217114 PMC5015806

[B47] Nagamatsu L. S. Chan A. Davis J. C. Beattie B. L. Graf P. Voss M. W. (2013). Physical activity improves verbal and spatial memory in older adults with probable mild cognitive impairment: a 6-month randomized controlled trial. *J. Aging Res.* 2013 1–10. 10.1155/2013/861893 23509628 PMC3595715

[B48] Ouellet M. Percival M. D. (2001). Mechanism of acetaminophen inhibition of cyclooxygenase isoforms. *Arch. Biochem. Biophys.* 387 273–280. 10.1006/abbi.2000.2232 11370851

[B49] Pickering G. Estève V. Loriot M.-A. Eschalier A. Dubray C. (2008). Acetaminophen reinforces descending inhibitory pain pathways. *Clin. Pharmacol. Ther.* 84 47–51. 10.1038/sj.clpt.6100403 17957182

[B50] Pickering G. Kastler A. Macian N. Pereira B. Valabregue R. Lehericy S. (2015). The brain signature of paracetamol in healthy volunteers: a double-blind randomized trial. *Drug Des. Dev. Ther.* 9 3853–3862. 10.2147/DDDT.S81004 26229445 PMC4517518

[B51] Polich J. (2007). Updating P300: an integrative theory of P3a and P3b. *Clin. Neurophysiol.* 118 2128–2148. 10.1016/j.clinph.2007.04.019 17573239 PMC2715154

[B52] Randles D. Heine S. J. Santos N. (2013). The common pain of surrealism and death. *Psychol. Sci.* 24 966–973. 10.1177/0956797612464786 23579320

[B53] Randles D. Kam J. W. Y. Heine S. J. Inzlicht M. Handy T. C. (2016). Acetaminophen attenuates error evaluation in cortex. *Soc. Cogn. Affect. Neurosci.* 11 899–906. 10.1093/scan/nsw023 26892161 PMC4884318

[B54] Roberts I. D. Krajbich I. Cheavens J. S. Campo J. V. Way B. M. (2018). Acetaminophen reduces distrust in individuals with borderline personality disorder features. *Clin. Psychol. Sci.* 6 145–154. 10.1177/2167702617731374

[B55] Sheth R. Marcon L. Bastida M. F. Junco M. Quintana L. Dahn R. (2012). Hox genes regulate digit patterning by controlling the wavelength of a turing-type mechanism. *Science* 338 1476–1480. 10.1126/science.1226804 23239739 PMC4486416

[B56] Smallwood J. Beach E. Schooler J. W. Handy T. C. (2008). Going AWOL in the brain: mind wandering reduces cortical analysis of external events. *J. Cogn. Neurosci.* 20 458–469. 10.1162/jocn.2008.20037 18004943

[B57] Smallwood J. Brown K. S. Tipper C. Giesbrecht B. Franklin M. S. Mrazek M. D. (2011). Pupillometric evidence for the decoupling of attention from perceptual input during offline thought. *PLoS One* 6:e0018298. 10.1371/journal.pone.0018298 21464969 PMC3064669

[B58] Sonuga-Barke E. J. S. Castellanos F. X. (2007). Spontaneous attentional fluctuations in impaired states and pathological conditions: a neurobiological hypothesis. *Neurosci. Biobehav. Rev.* 31 977–986. 10.1016/j.neubiorev.2007.02.005 17445893

[B59] Sridharan D. Levitin D. J. Menon V. (2008). A critical role for the right fronto-insular cortex in switching between central-executive and default-mode networks. *Proc. Natl. Acad. Sci. U.S.A.* 105 12569–12574. 10.1073/pnas.0800005105 18723676 PMC2527952

[B60] van Dinteren R. Arns M. Jongsma M. L. A. Kessels R. P. C. (2014). P300 development across the lifespan: a systematic review and meta-analysis. *PLoS One* 9:e0087347. 10.1371/journal.pone.0087347 24551055 PMC3923761

